# Maternal A90V mutation in the PreS1 gene of sub-genotype C2 hepatitis B virus is associated with intrauterine transmission

**DOI:** 10.1590/S1678-9946202365046

**Published:** 2023-09-08

**Authors:** Linzhu Yi, Jiaxin Wu, Zhiqing Yang, Yandi Li, Jia Lian, Tian Yao, Shuying Feng, Bo Wang, Yongliang Feng, Suping Wang

**Affiliations:** 1 Shanxi Medical University, Department of Epidemiology, Taiyuan, Shanxi, China; 2 Shanxi Medical University, Center of Clinical Epidemiology and Evidence Based Medicine, Taiyuan, Shanxi, China; 3 Shanxi Provincial Center for Disease Control and Prevention, Taiyuan, Shanxi, China; 4 Third People’s Hospital, Department of Obstetrics and Gynaecology, Taiyuan, Shanxi, China

**Keywords:** Hepatitis B virus, Mutation, Intrauterine transmission, PreS/S regions

## Abstract

PreS/S gene mutations could impact virus secretion, infection and immune evasion. However, the relationship between PreS/S mutations and intrauterine transmission has not yet been clarified. Thus, we aimed to explore the associations between PreS/S gene mutations of HBV isolated from mothers and intrauterine transmission. We analyzed the mutations of PreS/S regions of the HBV genome in mothers with HBV DNA levels ≥ 10^6^ IU/mL whose neonates experienced HBV intrauterine transmission (transmission group, GT) and those whose neonates did not experience intrauterine transmission (control group, GC) analyzed using clone-based sequencing. In total, 206 sequences were successfully amplified, including 98 sequences (from 21 mothers) from GT and 108 sequences (from 20 mothers) from GC of genotype C for mutational analysis. Among the 1203 nucleotides of PreS/S regions, there were 219 (18.20%) base substitutions, of which 103 (47.03%) base mutations caused amino acid changes. F80S, A90V and I68T were mutation hotspots. Mothers in GT had a higher mutation rate of A90V in the PreS1 gene than mothers in GC. The A90V mutation increased the risk of HBV intrauterine transmission after adjusting the maternal age and the mode of delivery (*OR* = 6.23, 95% *CI*: 1.18–32.97). Moreover, the area under the ROC curve (AUC) for intrauterine transmission due to A90V and a combination of A90V with the mode of delivery were 0.723 (95% *CI*: 0.575 to 0.891, *P* = 0.011) and 0.848 (95% *CI*: 0.723 to 0.972, *P* < 0.001), respectively. Mothers with the A90V mutation in the PreS1 gene may be a potential risk factor for HBV intrauterine transmission.

## INTRODUCTION

Approximately 86 million people in China and 296 million people worldwide are infected with the hepatitis B virus (HBV)^1,2^. Mother-to-child transmission (MTCT) is the main cause of chronic HBV infection^1^. There are three types of MTCT (intrauterine, intrapartum, and postpartum). Intrapartum and postpartum transmission have been effectively controlled by the administration of the hepatitis B vaccine and hepatitis B immunoglobulin (HBIG). However, these strategies have had a limited effect on reducing intrauterine transmission, which remains a major source of the reservoir of chronic HBV infection^3^. The incidence of HBV intrauterine transmission in China ranges from 3.73%-43.33%^4-6^, with 95% of neonates^7^ more likely to develop into chronic carriers, leading to a significant public burden. Consequently, to achieve the target of a 95% reduction in the incidence of HBV by 2030, more effective hepatitis elimination strategies and plans need to be developed^1^.

It has been reported that HBV has a high mutation rate due to its lack of a proofreading function for viral polymerase and the significantly increased pressure from host immune responses, leading to sequence heterogeneity^8,9^. HBV contains four partially overlapping open reading frames (ORFs): S ORF, C ORF, P ORF, and X ORF. The S ORF includes the Pre-S1, Pre-S2, and S genes, which encode three envelope proteins termed large (L), middle (M), and small (S) proteins (Supplementary Figure S1). The protein encoded by the HBV PreS/S regions plays an important role in the HBV life cycle^10^. As documented in studies, PreS/S gene mutations can directly impact virus secretion, infection, immune evasion, occult infection, resistance to drug therapy, and tolerance of immunoprophylaxis^11-14^. However, few studies have been conducted on PreS/S mutations and intrauterine transmission. Recently, five studies^15-19^ have demonstrated that some mutations in the HBV genome, such as P21L (PreS1 gene), P110S (PreS1 gene), P36L (PreS2 gene), C107R (S gene), have been detected, suggesting an association with the intrauterine transmission. More extensive studies on the association between HBV PreS/S mutation and intrauterine transmission would be important to further uncover the mechanisms of HBV intrauterine transmission.

This study analyzed the relationship between maternal HBV mutations (base substitutions [mutation hotspots and non-hotspots], deletions and insertions) in the PreS/S regions and neonatal HBV intrauterine transmission. This study aimed to identify new virological markers and provide evidence for new measures to reduce the risk of HBV infection in neonates.

## MATERIALS AND METHODS

### Subjects

The initial inclusion criteria were HBsAg positivity and a single pregnancy. We recruited 399 HBsAg-positive pregnant women who underwent prenatal examinations and delivered babies between June 2011 and July 2013 at the Department of Obstetrics and Gynecology, at the Third People’s Hospital in Taiyuan (37°, 54’ east longitude and 112°, 33’ north latitude). Informed consent was obtained from all human adult participants and parents or legal guardians of minors, along with approved consent of the Ethical Commission.

**Figure 1:**
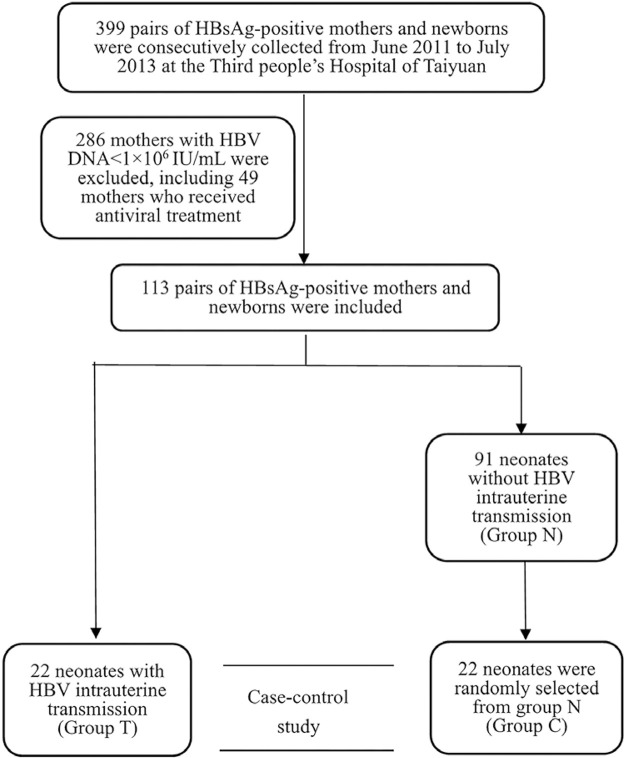
The flow chart of the selected subjects.

A total of 113 mothers with HBV DNA levels ≥ 10^6^ IU/mL were included. To avoid antiviral treatment-related effects on HBV mutation, those who began antiviral therapy during or before pregnancy were excluded. Twenty-two neonates with HBV intrauterine transmission were categorized into Group T (GT), and 91 neonates without HBV intrauterine transmission were categorized into Group N (GN). Subsequently, simple random sampling was performed to randomly select 22 neonates from Group N, which were categorized into Group C (GC) using SAS 9.4. No significant differences in general and viral characteristics were observed between Groups C and N. The sample selection process is shown in Figure 1. General demographic information was collected through face-to-face interviews and case inquiries. Three milliliters of maternal peripheral blood were collected before delivery, and neonatal femoral venous blood was collected within 24 h after birth (prior to passive-active immunoprophylaxis), and the sera were separated and stored at -80 °C for detection.

### Serological markers and serum HBV DNA assays

HBV serological markers were detected using electrochemiluminescence immunoassay (ECLIA) kits (Roche Diagnostics GmbH, Germany) and expressed as a COI (cut-off index). Serum HBV DNA levels were tested using a real-time PCR-TaqMan kit (DAAN Gene Co. Ltd, Sun Yat-sen University, Guangdong, China) and expressed as IU/mL. All procedures were performed according to the manufacturers’ instructions. HBsAg positive (> 1.00 COI) and/or HBV DNA positivity (> 100 IU/mL) for neonatal femoral blood samples were defined as HBV intrauterine transmission^5^.

#### HBV DNA extraction, amplification and sequencing

HBV DNA was extracted from 200 μL serum samples using the QIAamp DNA Mini kit (QIAGEN, Hilden, Germany). The PreS/S region (nt2848–nt835) was amplified using HBV-SF/SR primers and TransStart^®^ FastPfu DNA polymerase. The PCR procedure consisted of initial denaturation at 94 °C for five min, followed by 35 cycles of 94 °C for 30 s, 55 °C for 30 s, and 68 °C for 110 s, with a final extension of 10 min at 68 °C. Then, PCR products were validated by electrophoresis, and the favorable products were purified using the Gel Extraction Kit (OMEGA Biotek, Norcross, USA). These products were cloned into pEASY^®^-Blunt Zero vectors and transformed into Trans1-T1 Phage Resistant Chemically competent cells (TransGen Biotech, Beijing, China) which were grown on ampicillin plates. Positive clones were identified by PCR assay and then sequenced by Sangon Biotech Co., LTD (Shanghai, China). All primers are listed in Supplementary Table S1.

#### HBV genotype and sub-genotype

Standard sequences for HBV genotypes A-H, sub-genotypes B1-B6, and C1-C6 (Supplementary Tables S2-S4) from NCBI were used to identify the genotype and sub-genotype via Mega6.0. Phylogenetic analysis was performed by the Neighbor Joining method (Kimura 2-parameter Model, 1000 replicates).

## Mutational analysis

The reference sequence is listed in Supplementary Table S5. The sample sequences were compared with the reference sequences using Mega 6.0. A nucleotide substitution in the sequence contrasting with the reference sequence was considered a point mutation. A site in all obtained genotype C sequences with a proportion of mutations greater than 10% was defined as a hotspot^20^. Finally, a case-control study was employed to analyze the association between PreS/S gene mutations and HBV intrauterine transmission.

## Statistical analysis

Data were analyzed using the SAS statistical package (Version 9.4, SAS Institute Inc, Cary, NC, USA). Continuous variables were presented as the mean and standard deviation (SD), or as the median and range, and were analyzed using Student’s t-test or Wilcoxon rank sum test. The qualitative variables were analyzed using a chi-square test or Fisher’s exact test. Unconditional logistic regression was used for multivariate analysis. The potential predictive values were analyzed using receiver operating characteristic curve (ROC curve) analysis with the Statistical Package for Social Science (SPSS) for Windows, Version 22.0 (SPSS Inc., Chicago, IL, USA).

## RESULTS

### Incidence of HBV intrauterine transmission in HBsAg-positive mothers and neonates

A total of 399 pairs of HBsAg-positive mothers and neonates were consecutively collected from June 2011 to July 2013 at the Third People’s Hospital of Taiyuan. The incidence of HBV intrauterine transmission was 13.78% (55/399).

#### HBV genotypic distributions and relations to HBV intrauterine transmission

A total of 222 HBV sequences were obtained, including 104 sequences from 22 GTs and 118 sequences from 22 GCs. The mean ± SD (range) number of clones per sample was 5.0 ± 1.2 (4–6). There was no difference in the number of clones between the GT and GC groups (4.9 ± 1.4 vs. 5.2 ± 1.0, *P* = 0.764). The obtained sequences from the mothers were genotyped using standard sequences.

Genotype C accounted for the largest proportion, approximately 88.64% (39/44). Three mothers (6.82%) were of genotype B and 2 (4.55%) were of intergenotype B/C. Further sub-genotype results showed that sequences of genotype B were all identified as sub-genotype B2, and sequences of genotype C were identified as sub-genotype C2. From the clone’s perspective, sequences of sub-genotype C2 constituted 92.79% (206/222) of the HBV clones, of which 98 clones were in GT, and 108 clones were in GC. Sub-genotype B2 constituted 7.21% (16/222), of which 6 clones were in GT, and 10 clones were in GC (Supplementary Table S6).

#### General and viral characteristics of HBsAg-positive mothers and neonates

A total of 21 HBsAg-positive mothers (98 clones) from GT and 20 HBsAg-positive mothers (108 clones) from GC of sub-genotype C2 were included in the subsequent analysis.

All 41 mothers’ HBeAg tests were positive. The median loads of maternal HBV DNA were 9.57×10^7^ IU/mL and 4.94 ×10^7^ IU/mL in GT and GC, respectively (*P* = 0.657). There was a significant difference in the distribution of the mode of delivery between the two groups (*P* < 0.001), but no significant differences were observed in other characteristics between GT and GC (*P* > 0.05). The general and viral characteristics of these mothers and their neonates are summarized in Supplementary Table S7.

#### Relationship between mutations in the PreS/S regions of HBV subgenotype C2 and intrauterine transmission

To conduct a more accurate mutation analysis, the sequence of subgenotype C2 was used as the reference sequence. Sixty-three HBV standard sequences of the sub-genotype C2 from China in NCBI were downloaded. We used ClustalX 2.1 to align the sequences and import them into MegAlign software. According to the highest frequency of each point, the corresponding sequence was output to obtain the C2 reference sequence. Sample sequences were compared with the reference sequence to identify variations in the PreS/S regions of HBV genomes.

Among the 1,203 nucleotides in the PreS/S regions (Supplementary Figure S1), base substitutions and deletions were found, while no insertions were observed. Base substitution was the primary mutation, with a mutation rate of 18.20% (219/1,203). The mutation rate was lower in GT (6.57%, 79/1203) than in GC (14.46%, 174/1,203) (χ^2^ = 39.86, *P* < 0.001).

#### Relationship between base substitutions in the PreS/S regions of sub-genotype C2 and HBV intrauterine transmission

**Figure 2:**
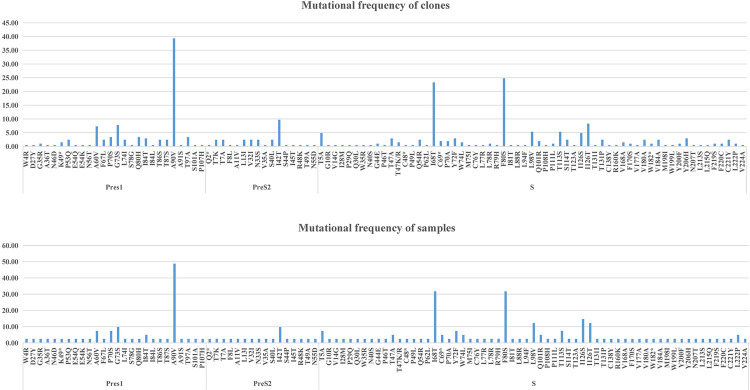
Mutation frequency (%) in the PreS/S regions of 206 sequences or 41 mothers.

There were 219 base substitutions in the PreS/S regions of HBV genotype C, with 103 base mutations causing amino acid changes (Supplementary Figure S2). The amino acid mutation rates of the samples and clones are detailed in Figure 2. We aggregated nonsynonymous mutation hotspots and non-hotspots in the PreS/S regions of HBV genotype C between GT and GC (Table 1). Among the 3 nonsynonymous mutation hotspots, F80S in the S gene was only present in GT. A90V in the PreS1 gene and I68T in the S gene were present in both groups. Whether from the mutation proportion of clones or samples, the mutation rate of A90V in GT was significantly higher in GT than in GC (*P* < 0.05, respectively), but no significant difference was observed in I68T. We also analyzed the non-hotspot mutations occurring in both groups, and the results are shown in Table 2. The mutation rates of G73S and I126T were higher in GC (*P* < 0.001, *P* = 0.002). Moreover, it should be pointed out that G73S and I126T/S were found in the major hydrophilic region (MHR) of HBV.

**Table 1:** Distribution of nonsynonymous mutations in the PreS/S regions of HBV sub-genotype C2.

Regions	Amino acid mutation type			

	Only in GT	Only in GC		Both
PreS1 (27)	D27Y	W4R	F67L	G73S
	N46D	G35R	S78G	I84T
	E54Q	A36T	Q80H	A90V^b^
	P70S	K49^a^	T86S	
	L74I	P53Q	A90I	
	I84L	E54K	A91S	
	T87S	N56T	T97A	
	S101A	A60V	P107H	
PreS2 (15)	Q2^a^	T7A/K	V32I	
	V35A	F8L	N33S	
	S44P	A11V	S40L	
	I45T	L13I	I42T	
	T49A			
	N55D			
S (61)	I28M	G10R	V177A	T5A
	P29Q	V14G	V180A	T47A^c^
	N40S^c^	Q30L	W182^a^	I68T^bc^
	C48^ac^	W35R^c^	Y200F	C69^ac^
	P70A^c^	G44E^c^	Y206H	L98V
	Y72F^c^	P46T^c^	N207T	I126S/T^c^
	M75I^c^	T47K/R^c^	L213S	
	C76Y^c^	Q54R^c^	L215Q	
	L77R	P62L^c^	F219S	
	F80S^b^	W74L^c^	F220C	
	I81T	R78L	C221Y	
	L94F	R79H	V224A	
	P108H^c^	L88R		
	T113S^c^	Q101R^c^		
	T123A^c^	P111L^c^		
	C138Y^c^	S114T^c^		
	V184A	T131I/P^c^		
	M198I	R160K^c^		
	W199L	V168A		
	L222P	F170S		

^a^Stop codon; ^b^Mutation hotpot; ^c^Occurred in MHR (aa 32-76 and aa 100-160 of S gene)

**Table 2:** Relationship between mutations in the PreS/S regions of HBV sub-genotype C2 and HBV intrauterine transmission.

Mutation	Region	Amino acid	Sample			Clone		
	
			GT n (%)	GC n (%)	*P*-value	GT n (%)	GC n (%)	*P*-value
Mutation hotspots	Pre-S1	A90V	14(66.67)	6(30.00)	<0.001^ad^	53(54.08)	28(25.93)	<0.001^d^
	S	F80S	13(61.90)	-^c^	-	51(52.04)	-^c^	
	S	I68T	7(33.33)	6(30.00)	0.819^b^	20(20.41)	28(25.93)	0.350^b^
Mutation non-hotspots	Pre-S1	G73S	1(4.76)	3(15.00)	0.552^a^	1(1.02)	15(13.89)	<0.001^ad^
	Pre-S1	I84T	1(4.76)	1(5.00)	0.371^a^	5(5.10)	1(0.93)	0.111^a^
	S	T5A	1(4.76)	2(10.00)	0.606^a^	3(3.06)	7(6.48)	0.338^a^
	S	T47A	1(4.76)	1(5.00)	0.371^a^	1(1.02)	5(4.63)	0.215^a^
	S	C69d	1(4.76)	1(5.00)	0.371^a^	1(1.02)	3(2.78)	0.623^a^
	S	L98V	3(14.29)	2(10.00)	1.000^a^	6(6.12)	5 (4.63)	0.760^a^
	S	I126T	2(9.52)	3(15.00)	1.000^a^	2(2.04)	15(13.89)	0.002^d^
	S	I126S	3(14.29)	3(15.00)	1.000^a^	3(3.06)	7(6.48)	0.338^a^

^a^Fisher’s exact test; ^b^Chi-square test; ^c^No mutations were found; ^d^ *P* < 0.05

A90V was introduced as a two-category variable into an unconditional logistic regression model to further analyze its impact on HBV intrauterine transmission. After adjusting for maternal age and the mode of delivery, maternal A90V mutation influenced HBV intrauterine transmission (Table 3). We also analyzed the relationships between A90V and HBV intrauterine transmission in neonates born by vaginal delivery or cesarean section separately. Maternal A90V mutation remained an influencing factor for HBV intrauterine transmission.

**Table 3:** Multivariate analysis of HBV intrauterine transmission.

Factors	N(%)	*OR* (95% *CI*)	Adjusted *OR* ^a^(95% *CI*)
C3116T (A90V)			
Non-mutation	21 (51.22)	1.00	1.00
Mutation	20 (48.78)	4.67 (1.25–17.43)^b^	6.23 (1.18–32.97)^b^
Mode of delivery			
Vaginal delivery	22 (53.67)	1.00	1.00
Cesarean section	19 (46.34)	0.08 (0.02–0.35)^b^	0.10 (0.02–0.48)^b^

^a^Adjusted factors: maternal age; ^b^ *P* < 0.05.

**Figure 3:**
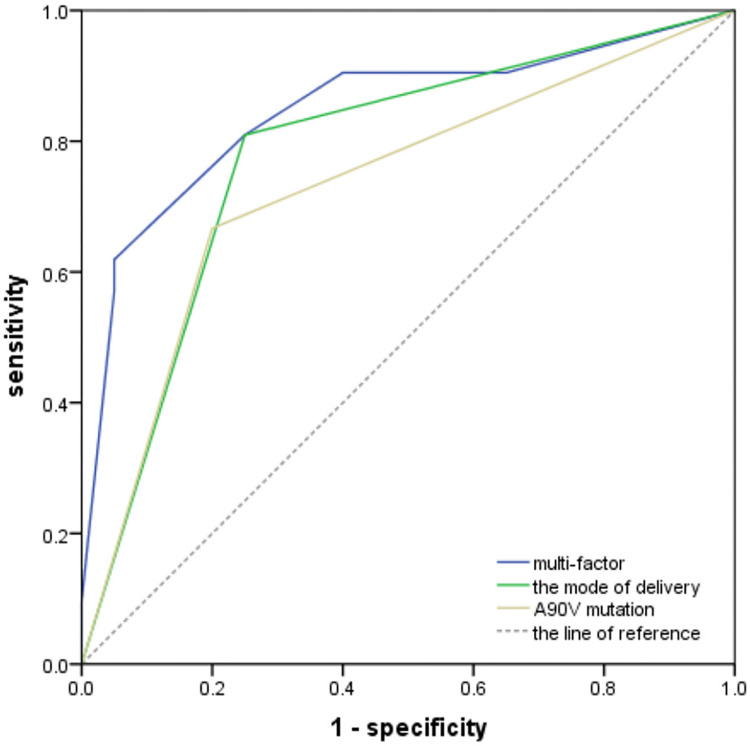
The predictive values of A90V mutation in the PreS1 gene and the mode of delivery for intrauterine transmission were analyzed by ROC curves. Multi-factor: a combination A90V mutation and the mode of delivery.

Next, the potential predictive values of A90V and the mode of delivery for intrauterine transmission were analyzed by ROC curve. As shown in Figure 3, the area under the ROC curve (AUC) for intrauterine transmission due to the A90V, the mode of delivery and a combination A90V with the mode of delivery were 0.723 (95% *CI*: 0.575 to 0.891, *P* =0.011), 0.780 (95% *CI*: 0.631 to 0.928, *P* = 0.002) and 0.848 (95% *CI*: 0.723 to 0.972, *P* < 0.001). These results suggested that A90V in the PreS1 gene and mode of delivery could predict the risk of intrauterine transmission for neonates in pregnant women with high HBV DNA load.

Furthermore, it was observed that the median level of HBsAg in neonates born with a maternal A90V mutation was higher than in those without an A90V mutation (1.43 COI vs. 0.62 COI, *Z* = 2.42, *P* = 0.018, Wilcoxon rank sum test). These results demonstrated that maternal A90V mutation in the PreS1 gene may be associated with a greater risk of HBV intrauterine transmission.

#### Relationship between deletion in the PreS/S regions of HBV sub-genotype C2 and HBV intrauterine transmission

Deletions were observed in both GT and GC, with no significant difference in the deletion rate between the two groups (5.10% vs. 9.26%, *P* = 0.293, Fisher’s exact test). The results of deletion were summarized in Supplementary Table S8. The 12 to 18 bp (12 to 21 bp) deletion in the PreS1 gene was found in both GT and GC, no significant difference was observed (4.63% vs. 4.08%, *P* = 1.000, Fisher’s exact test). It is noteworthy that five clones began at nt 3,016 with 183 bp of length were only found in GC.

## DISCUSSION

In this study, we investigated the relationship between the virus mutation in mothers with HBV DNA levels ≥10^6^ IU/mL and intrauterine transmission of neonates. We found that sequences of genotype C constituted the majority, which was consistent with the distributions reported in Northern China^21^. Additionally, the presence of intergenotype B/C also indicated a common phenomenon where different HBV genotypes could infect the same host.

All enrolled pregnant women did not receive any antiviral treatment so the influence of drugs could be excluded in our study. Previous studies have shown that neonates born to mothers who are HBeAg positive or have a high HBV DNA load are more likely to experience HBV intrauterine transmission^22^. However, there were no significant differences in HBeAg positive rates and HBV DNA levels between the two groups, so HBV sequences could be better used to explain the virological mechanism that leads to intrauterine transmission in neonates. Our study identified base substitutions and deletions, with the mutation rate of base substitutions in GT (6.57%) significantly lower than in GC (14.46%). Su *et al*.^16^ performed the same research and reached the same conclusion, suggesting that more mutations in GC might be advantageous to prevent HBV intrauterine transmission. Furthermore, our study revealed three mutation hotspots (A90V, F80S and I68T) causing amino acid changes in the HBV PreS/S regions of genotype C. F80S in the S gene was previously unreported and was found to be more prevalent in neonates with intrauterine transmission, suggesting a potential role in promoting intrauterine transmission. There was no significant difference in I68T between the two groups, suggesting that I68T in the S gene might not be closely associated with intrauterine HBV transmission. Nevertheless, a previous study^23^ showed that I68T in the S gene was an independent risk factor for hepatocellular carcinoma (HCC) development. Consequently, further studies on the potential role of I68T in the S gene are needed. The A90V mutation, located in the PreS1 gene (encoding large-HBsAg), was present in both GT and GC, with a higher mutation rate of A90V in GT than in GC. In addition, the A90V mutation was associated with an increased risk of HBV intrauterine transmission. This view differed from Cheng *et al*.^17^, whose results revealed that maternal A90V occurred less frequently in the HBV intrauterine infection group. The different sample sizes and the different levels of HBV DNA and HBeAg in the two studies might be the reason. However, some studies^20,24-26^ suggested that A90V was independently associated with an elevated risk of HCC, and A90V alone may be a predictive factor for the development of HCC during chronic HBV infection. It is speculated that A90V was located in the PreS1 region, where the mutations may be linked with viral retention and DNA oxidative damage^27^. Furthermore, A90V was located in the S promoter region (nt 2,809~3,152), which contains a heat-shock protein 70 (Hsc70) binding site and cytosolic anchorage determinant (CAD) that are vital for the dual topology of L proteins^28^. Additionally, amino acids aa58 to aa100 of PreS1 gene are known to be recognized by antibodies involved in viral clearance^29^. Notably, A90V was also detected in the same pair of mothers and neonates^17^, suggesting that the A90V of HBV from neonates originated from maternal transmission. Moreover, the ROC curve and the logistic regression analysis revealed that A90V and the mode of delivery were the potential predictors for neonates at a high risk of intrauterine transmission, thus suggesting A90V as a potential genetic marker for HBV intrauterine transmission risk.

Apart from mutation hotspots, significant differences in mutation non-hotspots (G73S, I126T) were observed between GT and GC in our study. The mutation rates of G73S and I126T were both higher in GC. G73S in the S promoter region was a novel mutation, suggesting that G73S mutation may reduce the expression of S protein and may hamper intrauterine transmission. The mutation rates of I126T in the current study were higher in GC than in GT. I126T has been reported previously^30,31^ and located in the “a” determinant region between amino acids 124–147. Mutations in the MHR, especially in the “a” determinant, may lead to the production of an antigenically modified S protein^12^. Some scholars^32,33^ have argued that the mutation on aa126 was within the first loop of the MHR and variants might impact HBsAg antigenicity. Furthermore, I126T is also located in the RT region overlapping with the S region. It has been reported that patients with RT and S substitutions had an overall significantly low HBsAg level, which may be the potential reason that mutation I126T was less frequent in GT than in the GC group in our study^34,35^. Notably, the mutation in the RT gene corresponding to the I126S in the S gene is D134E, which has been associated with treatment failure with tenofovir disoproxil fumarate^36^. Therefore, these findings suggest that I126T/S in the S gene are important mutations and might act as protective factors against intrauterine transmission. In addition, our study, except for I126T/S, identified a total of 28 mutations causing amino acid changes in the MHR, including N40S, G44E, Q54R, P70A, C76Y, I81T, Q101R, S114T and V168A, which have been reported in previous studies^31,37,38^. There still exist mutations causing amino acid changes that have not been reported previously in the MHR, and these mutations may affect HBsAg detection in the MHR, especially in the “a” region (aa 124–147). However, except for I126T, no significant difference was observed in the MHR between GT and GC. Given the region’s importance, this relationship should be a consideration for future long-term studies.

Moreover, some data showed that deletions in the PreS/S regions were found and may influence the amounts of circulating HBsAg^39^. The deletion of 183 bp (from nucleotides 3,019–3,201), which reduced the expression of HBsAg and secretion of viral particles^40^, was found in the GC rather than in GT, suggesting that deletion of 183 bp might block HBV intrauterine transmission. Some scholars also investigated the *in vitro* phenotype of viral strains with a 183-nucleotide deletion in the PreS1 region, resulting in the loss of the S promoter. Their findings revealed that mutations of a 183-nucleotide deletion led to a significant reduction of HBsAg secretion and a less efficient virion secretion^39^. Therefore, viral with a 183-nucleotide deletion in the PreS1 region could contribute to reduced intrauterine transmission.

Furthermore, it was reported^30^ that nucleotide sequence homology was 99.5–100% in the S gene between mothers and neonates. This point supports the importance of maternal mutations for HBV nucleotide sequences. However, our study has some limitations that need addressing. First, it would be better to have data on HBV mutations from neonates. Due to the low load of HBV DNA and the small amount of femoral venous blood collected from neonates, they were insufficient to sequence, and sequencing data from the neonates were not collected. Our study primarily focused on the role of maternal mutations in intrauterine transmission. Second, further large-scale multi-center prospective studies and relevant verification experiments are warranted to corroborate our findings in the future.

## CONCLUSION

In conclusion, we found some significant results, such as A90V, F80S, G73S, I126S/T and the 183bp deletion. Currently, preventive measures against neonatal HBV infection mainly target pregnant women. Therefore, prenatal detection of whether the mother carries the mutations of A90V in the PreS1 gene may offer predictive insight into neonatal HBV infection.
